# Taxonomic changes in *Oenothera* sections *Gaura* and *Calylophus* (Onagraceae)

**DOI:** 10.3897/phytokeys.28.6143

**Published:** 2013-11-04

**Authors:** Warren L. Wagner, Kyra N. Krakos, Peter C. Hoch

**Affiliations:** 1 Department of Botany, MRC-166, National Museum of Natural History, Smithsonian Institution, P.O. Box 37012, Washington, DC 20013-7012, USA; 2 Biology Department, Maryville University, 650 Maryville University Dr., St. Louis, MO 63141, USA; 3 Missouri Botanical Garden, P.O. Box 299, St. Louis, MO 63166-0299, USA

**Keywords:** *Oenothera*, *Gaura*

## Abstract

The long-recognized genus *Gaura* was shown recently to be deeply nested within one of two major clades of *Oenothera*. New molecular data indicate further taxonomic changes are necessary in *Oenothera* sect. *Gaura*. We make these changes here, including three new combinations, in advance of the Onagraceae treatment for the *Flora of North America*. The new phylogenetic studies show that several pairs of taxa treated as subspecies in the most recent revision by Raven and Gregory (1972) had independent origins within sect. *Gaura*, and are here elevated to species level (*Oenothera nealleyi* for *Gaura suffulta* subsp. *nealleyi*; *Oenothera dodgeniana* for *Gaura neomexicana* subsp. *neomexicana*; and *Oenothera podocarpa* for *Gaura hexandra* subsp. *gracilis*). Also, a nomenclatural problem in *Oenothera* sect. *Calylophus* is corrected by adopting the name *Oenothera capillifolia* Scheele for the species known previously, and nomenclaturally correct, as *Calylophus berlandieri* Spach. This problem necessitates a new combination *Oenothera capillifolia* subsp. *berlandieri*.

## *Oenothera* sect. *Gaura*

**Figure 1.**
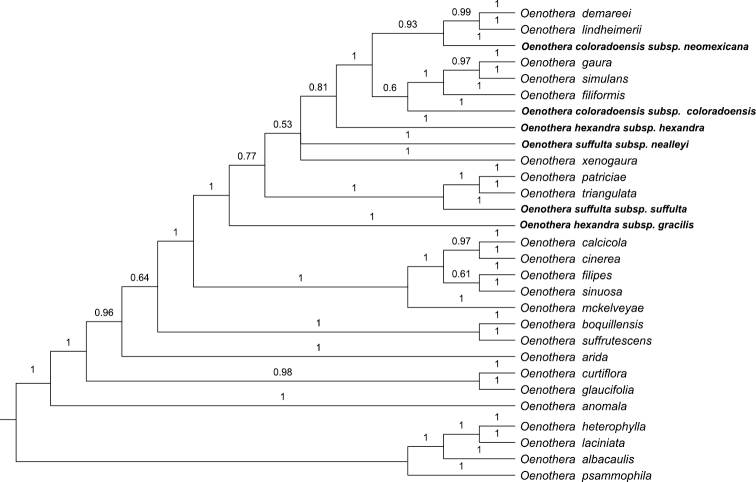
Portion of a Bayesian tree of *Oenothera* sect. *Gaura* within a larger analysis of one of two major clades of *Oenothera*, which has been referred to as clade B by Wagner et al. (2007) with several outgroup species of the other major clade (A) of *Oenothera* from Krakos et al. (unpubl.). The phylogeny is based on nuclear sequences of *ITS* and *ETS* and the chloroplast markers *rps*16, *ndh*F, *trn*L-F, and *rbcl*. The labels use the most recent prior taxonomy, placing the group in *Oenothera* (Wagner et al. 2007), using the taxon delimitations of Raven and Gregory (1972). The taxa involved in the proposed changes here are in bold. Numbers above nodes indicate Bayesian posterior probability values.

The molecular studies by Hoggard et al. (2004) and Levin et al. (2004) showed that the genus *Gaura* L. is deeply nested within *Oenothera* L. Wagner et al. (2007) in an overall revision of the Onagraceae at the generic and sectional level transferred the species of *Gaura* into *Oenothera* and made the group one of 18 sections of *Oenothera*. *Oenothera* sect. *Gaura* (L.) W. L. Wagner & Hoch is subdivided into eight subsections, with *Oenothera* subsect. *Gaura* (L.) W. L. Wagner & Hoch, which consists of 10 species (13 taxa) as delimited by Wagner et al. (2007), shown to be monophyletic by Hoggard et al. (2004) and Levin et al. (2004). Subsequent analyses by Krakos (2011; unpubl.; Fig. 1), which sampled all 13 taxa of subsect. *Gaura*, showed that none of the three species (*Oenothera hexandra*, *Oenothera suffulta*, and *Oenothera coloradensis*) subdivided into subspecies by Raven and Gregory (1972) and maintained in *Oenothera* by Wagner et al. (2007) were monophyletic. The relevant topologies revealed by Krakos (unpubl.) that indicate separate origins for these taxa are: 1) a strongly supported clade consisting of *Oenothera suffulta suffulta*, *Oenothera patriciae* W. L. Wagner & Hoch, and *Oenothera triangulata* (Buckley) W. L. Wagner & Hoch, but excluding *Oenothera suffulta nealleyi*, which is in a weakly supported grade sister to the *Oenothera suffulta suffulta* clade; 2) *Oenothera hexandra hexandra* is the first branch of a well-supported clade consisting of *Oenothera demareei* (P. H. Raven & D. P. Gregory) W. L. Wagner & Hoch, *Oenothera lindheimerii* (Engelmann & A. Gray) W. L. Wagner & Hoch, *Oenothera coloradensis neomexicana*, *Oenothera gaura* W. L. Wagner & Hoch, *Oenothera simulans* (Small) W. L. Wagner & Hoch, *Oenothera filiformis* (Small) W. L. Wagner & Hoch, and *Oenothera coloradensis coloradensis*, whereas *Oenothera hexandra gracilis* is sister to this clade plus the *Oenothera suffulta suffulta* clade; and 3) *Oenothera coloradensis coloradensis* and *Oenothera coloradensis neomexicana*, come out well-supported in different parts of the *Oenothera hexandra hexandra* clade described in #2. These results are consistent with those of Hoggard et al. (2004); however, that study did not include either *Oenothera suffulta nealleyi* or *Oenothera hexandra gracilis*. These results are also consistent with those of Levin et al. (2004), which did include *Oenothera hexandra gracilis*, but not *Oenothera suffulta nealleyi*.

Below we provide the nomenclature, revised descriptions, and geographic and ecological ranges for all six species of subsect. *Gaura* involved in these changes. We also provide a key to all 13 species of subsect. *Gaura*.

### Key to species of *Oenothera* sect. *Gaura* subsect. *Gaura*

**Table:** 

1a	Flowers 3-merous; fruits 3-angled	2
1b	Flowers 4-merous; fruits 4-angled	5
2a	Plants usually unbranched proximally, 60–180 cm tall; fruit narrowly ellipsoid or ellipsoid; coastal plain from Florida to North Carolina	*Oenothera simulans*
2b	Plants usually branched proximally, 15–65 cm tall; fruits ellipsoid or ovoid	3
3a	Sepals strigillose to subglabrous; pollen ca. 50 % fertile; north-central Texas and Oklahoma	*Oenothera triangulata*
3b	Sepals strigillose to glandular puberulent; pollen 90–100 % fertile	4
4a	Sepals 3–10 mm; inflorescence usually glandular puberulent; Mexico and Guatemala	*Oenothera hexandra*
4b	Sepals 10–15 mm; inflorescence strigillose; Texas, Oklahoma, Louisiana, and Alabama	*Oenothera patriciae*
5a	Flowers opening near sunrise; plants clumped perennial, usually branching from the base, villous throughout and usually glandular puberulent in the distal parts; southeast Texas and Louisiana	*Oenothera lindheimeri*
5b	Flowers opening near sunset or rarely sunrise (*Oenothera demareei*); plants annual or biennial, branched or unbranched; villous, strigillose or short-hirtellous throughout, distal parts usually glandular puberulent, short-hirtellous, strigillose, or subglabrous, occasionally villous	6
6a	Fruits angled but not winged; plants (5–)100–400 cm tall	7
6b	Fruits broadly winged on the angles and deeply furrowed between the angles; plants rarely more than 15–100(–120) cm tall	12
7a	Sepals 2.5–8 mm; stem usually single, branched or unbranched from base upwards; coastal plain from Florida to North Carolina	*Oenothera simulans*
7b	Sepals (7–)9–20 mm; stems usually several, branched from base upwards	8
8a	Flowers opening near sunrise; plants exclusively strigillose throughout; southwestern Arkansas, and perhaps adjacent Texas and Oklahoma	*Oenothera demareei*
8b	Flowers opening near sunset; plants with some combination of villous, glandular puberulent, short-hirtellous, strigillose, or subglabrous; rarely exclusively strigillose	9
9a	Anthers with pollen 35–65% fertile (sterile pollen smaller); plants villous and glandular puberulent, never strigillose; north-central to north-eastern U.S.	*Oenothera gaura*
9b	Anthers with pollen 90–100% fertile; plants strigillose, villous, short-hirtellous, or subglabrous	10
10a	Plants annual; fruit 4.5–7 mm; central U.S.	*Oenothera filiformis*
10b	Plants biennial; fruit 6–11 mm; Colorado, Nebraska, New Mexico, and Wyoming	11
11a	Plants strigillose proximally, becoming glandular puberulent and strigillose distally; Colorado, Nebraska, and Wyoming	*Oenothera coloradensis*
11b	Plants villous and strigillose proximally, becoming also glandular puberulent distally and only sparsely villous; Colorado and New Mexico	*Oenothera dodgeniana*
12a	Sepals 11–20 mm; floral tube 7–20 mm	13
12b	Sepals 6–15 mm; floral tube 6–12 mm	14
13a	Plants glabrous distally, except sometimes bracts sparsely villous; fruit sessile	*Oenothera suffulta*
13b	Plants glandular puberulent distally; fruit on a stipe 0.2–2 mm	*Oenothera nealleyi*
14a	Sepals 6–12 mm long, glandular puberulent, subglabrous or strigillose; Mexico to Arizona, and New Mexico	*Oenothera podocarpa*
14b	Sepals 10–15 mm, glabrous or strigillose; Texas, Oklahoma, Louisiana, and Alabama	*Oenothera patriciae*

### 1. Oenothera nealleyi

(J. M. Coulter) Krakos & W. L. Wagner comb. nov.

urn:lsid:ipni.org:names:77133597-1

http://species-id.net/wiki/Oenothera_nealleyi

#### Basionym.

*Gaura nealleyi* J. M. Coulter, Contr. U.S. Natl. Herb. 1: 38. 1890. *Gaura suffulta nealleyi* (J. M. Coulter) P. H. Raven & Gregory, Mem. Torrey Bot. Club 23: 81. 1973 [“1972”]. *Oenothera suffulta* (Engelmann ex A. Gray) W. L. Wagner & Hoch subsp. *nealleyi* (J. M. Coulter) W. L. Wagner & Hoch, Syst. Bot. Monogr. 83: 214. 2007.

#### Type.

United States. Texas: Presidio County, Chenate [Chinati] region, 1889, G. C. Nealley 545 [the species entry number (150) from Coulter’s publication also on sheet below 545] (Holotype: US-00015158!; isotype: F!).

*Gaura suffulta* Engelmann ex A. Gray var. *terrellensis* Munz, Bull. Torrey Bot. Club 65: 121. 1928.

Type: United States. Texas: Terrell County, Sanderson, 1 May 1934, V. L. Cory 8469 (Holotype: POM-200873; Isotype: TAES).

Annual from a stout taproot, usually branched from the base, 20–70 (-100) cm tall, villous proximally, the leaves subglabrous or sparsely villous along the veins and margins, becoming glandular puberulent in the distal portion of the plant, especially on the outside of the floral tube and the sepals. Leaves: rosette leaves lyrate, 3.5–9 × 0.5–1.5 cm, gradually narrowed to the petiole, cauline leaves 1.5–7 × 0.1–0.6 cm, narrowly lanceolate to linear, margin sinuate-dentate, undulate, subsessile. Flowers 4-merous, opening at sunset; floral tube 10–20 mm; sepals 11–21 mm; petals 10–15 mm; staminal filaments 8–13 mm, anthers 2–6 mm, pollen 90–100% fertile; style 22–36 mm. Capsule indehiscent, 4.5–8 × 2–5 mm, nut-like, hard, woody, not reflexed, the body ellipsoid or ovoid, broadly winged on the angles and deeply furrowed between the angles, lower angles or projections of the wings at or above the middle prominent; stipe 0.2–2.2 mm long. Seeds 3–4 (-5), 2–3 (-4) mm, ovoid, usually flattened on one or several sides by crowding in the fruit, yellowish to light brown. Gametic chromosome number: n = 7. Self-incompatible.

#### Phenology and distribution.

Flowering from April to October. Trans-Pecos Texas and northern Coahuila, Mexico, north to Bernalillo and Torrance counties, New Mexico, in washes and other sandy places, grasslands, and extending to pinyon-juniper woodlands; 1220–2140 m.

Raven and Gregory (1972) considered *Oenothera nealleyi* to represent an unevenly intergrading entity with *Oenothera suffulta* based on geographical merging of some of the characteristics that distinguish them and because the entity from Terrell County, Texas, described as *Gaura suffulta* var. *terrellensis* Munz has a combination of characteristics of the two taxa. The origin of these plants from Terrell County should be further explored. For now we here continue to include this name in the synonymy of *Oenothera nealleyi*. The molecular data (Krakos 2011; unpubl.) suggest that *Oenothera nealleyi* is not as closely related to *Oenothera suffulta* as suggested by Raven and Gregory (1972), given the placement in the phylogeny and the difference in scent profiles for these two taxa. *Oenothera suffulta* is a member of a strongly supported clade (Fig. 1) consisting of it along with *Oenothera triangulata* and *Oenothera patriciae*, while *Oenothera nealleyi* is a member of a polytomy that consists of other species of subsect. *Gaura*, with the *Oenothera suffulta* - *Oenothera triangulata* - *Oenothera patriciae* clade sister to it. *Oenothera nealleyi* has a strong sweet scent, characterized by benzaldehyde (almond), cinnamaldehyde, cinnamic alcohol (cinnamon), methyl salicylate and its methyl ether (wintergreen), neral and geranial (citronella), and nerol and geraniol (lemon) (R. Raguso, pers. comm.), whereas *Oenothera suffulta* does not have a discernible scent. This difference in scent probably plays a key role in the pollination syndromes for these species.

### 2. Oenothera suffulta

(Engelmann ex A. Gray) W. L. Wagner & Hoch, Syst. Bot. Monogr. 83: 214. 2007.

http://species-id.net/wiki/Oenothera_suffulta

#### Basionym.

*Gaura suffulta* Engelmann ex A. Gray, Bost. J. Nat. Hist. 6: 190. 1850.

#### Type.

United States. Texas: Comal County, New Braunfels, May 1847, F. Lindheimer 611 (Lectotype, designated by Raven & Gregory, 1972 [1973]: 80, GH- 00054125!; Isolectotypes: BM, F!, GH [3]!, K [2]!, LE, M!, MO [3]!, NY!, PH, TEX!,, US[2]!, YU!).

Annual from a stout taproot, moderately branched from the base, 25–120 cm tall, villous proximally, the leaves subglabrous or sparsely villous along the veins and around the margins, becoming glabrous distally or rarely sparsely villous on the bracts. Leaves: rosette leaves 7–11 × 0.1–2.3 cm, lyrate, gradually narrowed to the petiole; cauline leaves 1–9.5 × 0.1–2.3, narrowly lanceolate to linear, margin sinuate-dentate, undulate, subsessile. Flowers 4-merous, opening at sunset; floral tube 6.5–14 mm; sepals 11–21 mm; petals 10–15 mm; staminal filaments 6–9 mm, anthers 2–6 mm, pollen 90–100% fertile; style 16–32 mm. Capsule indehiscent, 4.5–8 × 2–5 mm, nut-like, hard, woody, not reflexed, the body ellipsoid or ovoid, broadly winged on the angles and deeply furrowed between the angles, without prominent lower corners or projections of the wings at or above the middle; stipe 0–1 mm long. Seeds (1-)2–4, 2–2.5 mm, ovoid, usually flattened on one or several sides by crowding in the fruit, yellowish to light brown. Gametic chromosome number: n = 7. Self-incompatible.

#### Phenology and distribution.

Flowering from April to June. Common in western Texas, but rare elsewhere throughout the state and absent in the Trans-Pecos; southern Oklahoma, east to Tulsa, Okfuskee, and Coal counties, and in Woodward County, in open, sandy places; 10–1010 m.

### 3. Oenothera dodgeniana

Krakos & W. L. Wagner nom. nov.

urn:lsid:ipni.org:names:77133475-1

#### Basionym.

*Gaura neomexicana* Wooton, Bull. Torrey Bot. Club 25: 307. 1898, non *Oenothera neomexicana* (Small) Munz (1931). *Oenothera coloradensis* (Rydberg) W. L. Wagner & Hoch subsp. *neomexicana* (Wooton) W. L. Wagner & Hoch, Syst. Bot. Monogr. 83: 211. 2007.

#### Type.

United States. New Mexico: Lincoln County, White Mts., 6500 ft, 25 Jul 1897, E. O. Wooton 204 (Holotype: US-330429!; Isotypes: AC!, E!, GH!, K!, LE, MIN, MO! ND, NY [2]!, P, US!).

Biennial from a stout fleshy taproot, with a single or a few branches from the base, 50–120 cm tall, villous and strigillose proximally, leaves subglabrous or strigillose, becoming also glandular puberulent distally, and sometimes also sparsely villous. Leaves: rosette leaves 6–20 × 1–3 cm; cauline leaves 5–10 × 1–2.5 cm, lanceolate to narrowly elliptic, subentire to repand-denticulate. Flowers 4-merous, opening at sunset; floral tube 10–11 mm; sepals 11–15 mm; petals 11–13.5 mm; staminal filaments 6.5–9 mm, anthers 2.5–4 mm long, pollen 90–100% fertile; style 22–28 mm. Capsule indehiscent, 9–11 × 3–5 mm, nut-like, hard, woody, not reflexed, the body ellipsoid or ovoid, sharply 4-angled, with fairly deep furrows alternating with the angles for 2–3 mm from the apex, ribbed from base of furrow to base of the fruit. Seeds 2–4, 2–3 mm, yellowish to light brown. Gametic chromosome number: n = 7. Self-compatible.

#### Phenology and distribution.

Flowering from June to September.In the western foothills of the San Juan Mountains in Archuleta County, Colorado, and Rio Arriba County, New Mexico; Sierra Blanca and Sacramento Mountains in Lincoln and Otero counties, south-central New Mexico. Collected once at Durango, La Plata County, Colorado (Raven and Gregory 1972), but has not since been recollected; found in mountain meadow openings in coniferous forests; 1830–2640 m.

The new name for this species is to honor David and Judy Dodgen of Cloudcroft, New Mexico, landowners who graciously allowed one of us (Krakos) to conduct research on their land, which harbors populations of this rare species. *Oenothera dodgeniana* and *Oenothera coloradensis* were considered by Raven and Gregory (1972) to represent a relict species along the eastern flank of the Rocky Mountains that arose from more widespread species farther to the east, such as *Oenothera filiformis*. The new molecular analyses revealed that *Oenothera coloradensis* is closely related to *Oenothera filiformis* (Fig. 1). *Oenothera coloradensis* is the first branch of a subclade within the subsect. *Gaura* clade that includes *Oenothera filiformis* and a terminal sister pair of *Oenothera gaura* and *Oenothera simulans*. These relationships suggest that *Oenothera coloradensis* may represent a relictual species and have a shared ancestry with *Oenothera filiformis*. *Oenothera dodgeniana*, on the other hand, belongs to a subclade which is sister to that containing *Oenothera coloradensis*, and within that subclade is sister to *Oenothera demareei* and *Oenothera lindheimeri*. So, although *Oenothera dodgeniana* is fairly closely related to *Oenothera coloradensis*, the two taxa seem to have had independent origins that have led to distributions along the eastern flank of the Rocky Mountains.

### 4. Oenothera coloradensis

(Rydberg) W. L. Wagner & Hoch, Syst. Bot. Monogr. 83: 211. 2007.

http://species-id.net/wiki/Oenothera_coloradensis

#### Basionym.

*Gaura coloradensis* Rydberg, Bull. Torrey Bot. Club 31: 572. 1904. *Gaura neomexicana* var. *coloradensis* (Rydberg) Munz, Bull. Torrey Bot. Club 65: 114. 1938. *Gaura neomexicana coloradensis* (Rydberg) P. H. Raven & Gregory, Mem. Torrey Bot. Club 23: 63. 1973 [“1972”].

#### Type.

United States. Colorado: Larimer County, Ft. Collins, 5000 ft, 8 Jul 1895, J. M. Cowen s.n. (Holotype: NY-BC00232160!; Isotype: GH!).

Biennial from a stout, fleshy taproot, with several branches from the base, 50–80(-100) cm tall, strigillose proximally, becoming glandular puberulent and strigillose distally. Leaves: rosette leaves 4–18 × 1.5–4 cm; cauline leaves 5–13 × 1–4 cm, very narrowly elliptic, subglabrous or strigillose, margin subentire to repand-denticulate. Flowers 4-merous, opening at sunset; floral tube 8–12 mm; sepals 9.5–13 mm; petals 7–12 mm; staminal filaments 6.5–9 mm, anthers 2.5–4 mm, pollen 90–100% fertile; style 19–25 mm. Capsule indehiscent, 6–8.5 × 2–3 mm, nut-like, hard, woody, not reflexed, the body ellipsoid or ovoid, sharply 4-angled, with fairly deep furrows alternating with the angles for 2–3 mm from the apex, ribbed from base of furrow to base of the fruit. Seeds 1–4, 2–3 mm, yellowish to light brown. Gametic chromosome number: n = 7. Probably self-compatible.

#### Phenology and distribution.

Flowering in July and August.In early successional vegetation of the North and South Platte River watersheds on the high plains, sloping floodplains, and drainage base in heavy soils, from southern Laramie and Platte counties in Wyoming, northern Weld County, Colorado, formerly near Fort Collins, Larimer County, Colorado, and in western Kimbal County, Nebraska; 1530–1950 m.

The Colorado butterfly plant is currently known from fewer than two dozen populations and has been federally listed as a Threatened species in the U.S. (USDI Fish and Wildlife Service 2000). The primary threats are agricultural use of habitat, herbicide spraying to control weed species, and livestock trampling and grazing (see Fertig 2000, Heidel and Handley 2011, NatureServe 2013). Recent study by Krakos (unpubl.) has determined this species to probably be self-compatible.

### 5. Oenothera podocarpa

(Wooton & Standley) Krakos & W. L. Wagner comb. nov.

urn:lsid:ipni.org:names:77133476-1

http://species-id.net/wiki/Oenothera_podocarpa

#### Basionym.

*Gaura podocarpa* Wooton & Standley, Contr. U.S. Natl. Herb. 16: 154. 1913.

#### Type.

United States. New Mexico: Grant County, Bear Mt., near Silver City, 1500 m, 17 June 1903, O. B. Metcalfe166 (Holotype: US-495277!; Isotypes: DS, E!, G, GH!, LE, MIN!, MO!, NMC! POM, US!).

### Oenothera hexandra (Ortega) W. L. Wagner & Hoch subsp. gracilis

(Wooton & Standley) W. L. Wagner & Hoch, Syst. Bot. Monogr. 83: 213. 2007.

#### Basionym.

*Gaura gracilis* Wooton & Standley, Contr. U.S. Natl. Herb. 16: 153. 1913. *Gaura hexandra gracilis* (Wooton & Standley) P. H. Raven & Gregory, Mem. Torrey Bot. Club 23: 87. 1973 [“1972”].

#### Type.

United States. New Mexico: Grant County, Ft. Bayard forest nursery, 29 Aug 1905, J. C. Blumer 44 (Holotype: US-499693!; isotypes: GH!). Epithet not available in *Oenothera* as there are two earlier uses: *Oenothera gracilis* (Phil.) H. Léveillé, 1905 (syn. for *Gayophytum micranthum* Hooker & Arnott) and *Oenothera gracilis* Schrader ex Fischer & Meyer, 1835 (syn. For *Oenothera perennis* L.).

### Gaura strigillosa

Wooton & Standley, Contr. U.S. Natl. Herb. 16: 154. 1913.

#### Type.

United States. New Mexico: Lincoln County, Wingfields Ranch on Ruidosa Creek, White Mts, 8 July 1895, E. O. Wooton s.n. (Holotype: US-561073!).

### Gaura brassicacea

Wooton & Standley, Contr. U.S. Natl. Herb. 16: 152. 1913.

#### Type.

United States. New Mexico: Socorro County, Socorro, May 1881, G.R. Vasey s.n. (Holotype: US-45764!).

### Gaura glandulosa

Wooton & Standley, Contr. U.S. Natl. Herb. 16: 153. 1913.

http://species-id.net/wiki/Gaura_glandulosa

#### Type.

United States. New Mexico: Catron County, Reserve, 9 July 1906, E. O. Wootons.n. (Holotype: US 561072!; Isotype: US!).

Annual herb from a stout taproot, usually well-branched at the base and above, 15–100 cm tall, villous proximally, the leaves subglabrous to densely short-villous, becoming subglabrous, strigillose and/or glandular puberulent distally. Leaves: rosette leaves lyrate, 3–15 × 0.5–1 cm; cauline leaves 1–9 × 0.1–0.8 cm, linear to very narrowly elliptic or narrowly lanceolate, sinuate-dentate to subentire, subsessile. Flowers 4-merous, opening at sunset; floral tube 6–10 mm; sepals 6–12 mm; petals 5.5–9.5 mm; filaments 4–6 mm, anthers 2–3 mm, pollen 90–100% fertile; style 11–18.5 mm. Capsule indehiscent, 6–8 × 2–3 mm, nut-like, hard, woody, not reflexed, broadly winged on the angles and deeply furrowed between the angles, the body ellipsoid or narrowly obovoid, narrowed at the base but not stipitate. Seeds 4, 2–3 mm, ovoid, usually flattened on one or several sides by crowding in the fruit, yellowish to reddish brown. Gametic chromosome number: n = 7. Self-compatible.

#### Phenology and distribution.

Flowering from (May) June to October. In Arizona from eastern Mohave County south through the mountains of central Arizona to eastern Pima County and the southwestern quarter of New Mexico, and in Mexico southward in the Sierra Madre Occidental to eastern Sonora and throughout the western halves of Chihuahua and Durango, often in disturbed sites in or on sandy washes, slopes, grasslands, meadows, pinyon-juniper or ponderosa pine woodlands, and sometimes on volcanic cinders; 760–2750 m.

*Oenothera podocarpa* is the first species to branch off in the subsect. *Gaura* clade (Fig. 1), whereas *Oenothera hexandra* is nested well within the subsect. *Gaura* clade. The epithet “podocarpa” was selected among the three equally available names at the species rank for this species. Previously, *Gaura gracilis*, one of four species published simultaneously by Wooton and Standley,was selected by Munz (1938), while placing the other three into synonymy. Tidestrom and Kittell (1941) apparently unaware of the Munz publication selected *Gaura glandulosa*, but were incorrect as they should have used *Gaura gracilis*. They did not establish any new priority.

### 6. Oenothera hexandra

(Ortega) W. L. Wagner & Hoch, Syst. Bot Monogr. 83: 212. 2007.

http://species-id.net/wiki/Oenothera_hexandra

#### Basionym.

*Gaura hexandra* Ortega, Hort. matr. dec. 14. 1797.

#### Type.

Based on living plants cultivated at the Royal Botanical Garden in Madrid from seeds sent by Sessé from Mexico [erroneously said to be from Cuba] (Holotype: not located).–Mexico. México. Comunidad Temascaltepec, 19 May 1936, G. B. Hinton 7688 (Neotype, designated, by Wagner et al. 2007: 212: MO-1717467!; Isoneotypes: C, F, G, GH, LL, MICH, NY, US!).

Annual herb from a stout taproot, usually well-branched at the base and above, 15–100 cm tall, villous proximally, the leaves subglabrous to densely short-villous, and becoming subglabrous, strigillose, and/or glandular-puberulent distally. Leaves in a basal rosette and cauline; rosette leaves lyrate, gradually narrowed to the petiole, usually quickly deciduous; cauline leaves 1–9 × 0.1–0.8 cm, linear to very narrowly elliptic or narrowly lanceolate, margin sinuate-dentate to subentire, subsessile. Inflorescence strict to somewhat branched, 7–53 cm long, bracts 2–5 cm long, narrowly lanceolate to ovate. Flowers 3-merous, opening at sunset; floral tube 4.5–7.5 mm; sepals 3–10 mm; petals 4.5–7 mm; staminal filaments 3–6 mm, anthers 1–2 mm, pollen 90–100% fertile; style 9–14.5 mm. Capsule indehiscent, 4.5–8 × 2–4.5 mm, nut-like, hard, woody, not reflexed, the body ellipsoid or narrowly obovoid, broadly winged on the angles and deeply furrowed between the angles, narrowed at the base but not stipitate. Seeds 3, 1.75–3 mm, ovoid, usually flattened on one or several sides by crowding in the fruit, yellowish to reddish brown. Gametic chromosome number: n = 7. Self-compatible and highly autogamous.

#### Phenology and distribution.

Flowering from March to November. From Durango, Mexico south in the Sierra Madre Occidental to the Trans Mexican Volcanic Belt, where abundant, and in Chiapas, Mexico as well as Guatemala in grasslands, meadows or oak woodlands, or disturbed areas, in sandy soils; 1800–2430 m.

## *Oenothera* sect. *Calylophus*

A nomenclatural problem in *Oenothera* sect. *Calylophus* is corrected here by adopting the name *Oenothera capillifolia* Scheele for the species known previously, and nomenclaturally correct, as *Calylophus berlandieri* Spach when the genus *Calylophus* is recognized as distinct from *Oenothera*; however, when this species is considered to be a member of *Oenothera* as it was recently by Wagner et al. (2007) based on molecular data (Levin et al. 2004), *Oenothera berlandieri* (Spach) Steudel is not available since it is a later homonym. This problem necessitates a new combination *Oenothera capillifolia berlandieri*.

### 7. Oenothera capillifolia

Scheele, Linnaea 21: 576. 1848.

http://species-id.net/wiki/Oenothera_capillifolia

#### Basionym.

*Meriolix capillifolia* (Scheele) Small, Fl. S.E. U.S. 846, 1335. 1903.

#### Type.

United States. Texas: Comal County, NewBraunfels, April (1846?), Ferdinand Roemers.n. (not located). —United States. Texas: Comal County, NewBraunfels, May 1850, F. Lindheimer 809 (Neotype: here designated, US-502186!; Isoneotypes: ARIZ, DS, F, GH, MO!, NMC, NY, OKL, PH, TEX, UC).

Towner (1977) subdivided *Oenothera capillifolia* into two subspecies. We here continue to use his classification with the new nomenclature.

### 7a. Oenothera capillifolia Scheele subsp. capillifolia

Oenothera berlandieri subsp. *pinifolia* (Engelmann) W. L. Wagner & Hoch, Syst. Bot Monogr. 83: 211. 2007.

#### Basionym.

*Oenothera serrulata* var. *pinifolia* Engelmann in A. Gray, Bost. J. Nat. Hist. 6: 189. 1850. *Meriolix serrulata* var. *pinifolia* (Engelmann) Small, Bull. Torrey Bot. Club 23: 187. 1896. *Oenothera serrulata pinifolia* (Engelmann) Munz, N. Amer. Fl., ser. 2, 5: 141. 1965. *Calylophus berlandieri pinifolius* (Engelmann) Towner, Ann. Missouri Bot. Gard. 64: 107. 1977.

#### Type.

United States. Texas: Comal County, New Braunfels, Apr-May 1846, F. Lindheimer 394 (Holotype: MO-122323!; Isotypes: DS, GH [2]!, K [3]! MO!, NY!, PH, RSA, US!, YU!).

### 7b. Oenothera capillifolia Scheele subsp. berlandieri

(Spach) W.L. Wagner & Hoch comb. nov.

urn:lsid:ipni.org:names:77133601-1

#### Basionym.

*Calylophus berlandieri* Spach, Ann. Sci. Nat. Bot., sér. 2, 4: 272. Nov. 1835. *Oenothera berlandieri* (Spach) Steud., Nom. Bot., ed. 2. 2: 206. 1841, non D. Dietr. Dec. 1840. *Meriolix berlandieri* (Spach) Walp., Repert. Bot. Syst. 2: 79. 1843. *Calylophus drummondianus* Spach subsp. *berlandieri* (Spach) Towner & Raven, Madroño 20: 243. 1970.

#### Type.

United States. Texas: Bahia del Espiritu Santo [probably in present Calhoun County, March or May 1829], Jean Louis Berlandier 539=1919 (Holotype: P; Isotypes: GH!, PH).

## Supplementary Material

XML Treatment for Oenothera nealleyi

XML Treatment for Oenothera suffulta

XML Treatment for Oenothera dodgeniana

XML Treatment for Oenothera coloradensis

XML Treatment for Oenothera podocarpa

XML Treatment for Oenothera hexandra (Ortega) W. L. Wagner & Hoch subsp. gracilis

XML Treatment for Gaura strigillosa

XML Treatment for Gaura brassicacea

XML Treatment for Gaura glandulosa

XML Treatment for Oenothera hexandra

XML Treatment for Oenothera capillifolia

XML Treatment for Oenothera capillifolia Scheele subsp. capillifolia

XML Treatment for Oenothera capillifolia Scheele subsp. berlandieri
